# Pretreatment platelet count improves the prognostic performance of the TNM staging system and aids in planning therapeutic regimens for nasopharyngeal carcinoma: a single-institutional study of 2,626 patients

**DOI:** 10.1186/s40880-015-0006-x

**Published:** 2015-03-05

**Authors:** Yu-Pei Chen, Bing-Cheng Zhao, Chen Chen, Lu-Jun Shen, Jin Gao, Zhuo-Yao Mai, Meng-Kun Chen, Gang Chen, Fang Yan, Su Liu, Yun-Fei Xia

**Affiliations:** Department of Radiation Oncology, State Key Laboratory of Oncology in South China; Collaborative Innovation Center for Cancer Medicine; Sun Yat-sen University Cancer Center, Guangzhou, Guangdong 510060 PR China; Zhongshan School of Medicine, Sun Yat-sen University, Guangzhou, Guangdong 510080 PR China; Department of Radiation Oncology, Anhui Provincial Hospital, Hefei, Anhui 230001 PR China; Department of Radiation Oncology, Sun Yat-sen University Cancer Center, 651 Dongfeng Road East, Guangzhou, Guangdong 510060 PR China

**Keywords:** Platelet count, Nasopharyngeal carcinoma, Prognosis, Neoplasm staging, Matched-pair analysis

## Abstract

**Introduction:**

Thrombocytosis has been identified as an unfavorable prognostic factor in several types of cancer. This study aimed to evaluate the prognostic value of pretreatment platelet count in association with the TNM staging system and therapeutic regimens in patients with nasopharyngeal carcinoma (NPC).

**Methods:**

A total of 2,626 patients with NPC were retrospectively analyzed. Platelet count >300 × 10^9^/L was defined as thrombocytosis. Matched-pair analysis was performed between patients receiving chemoradiotherapy and radiotherapy.

**Results:**

Multivariate analysis showed that platelet count was an independent unfavorable prognostic factor for overall survival (OS) [hazard ratio (HR) = 1.810, 95% confidence interval (CI) = 1.531–2.140, *P* < 0.001] and distant metastasis–free survival (DMFS) (HR = 1.873, 95% CI = 1.475–2.379, *P* < 0.001) in the entire patient cohort. Further subgroup analysis revealed that increased platelet count was an independent unfavorable prognostic factor for OS and DMFS in patients with NPC stratified by early and advanced T category, N category, or TNM classification (all *P* ≤ 0.001). Receiver operating characteristic (ROC) curves verified that the predictive value of TNM classification for OS was improved when combined with pretreatment platelet count (*P* = 0.030). Matched-pair analysis showed that chemoradiotherapy significantly improved OS only in advanced-stage NPC with thrombocytosis (HR = 0.416, 95% CI = 0.226–0.765, *P* = 0.005).

**Conclusions:**

Pretreatment platelet count, when combined with TNM classification, is a useful indicator for metastasis and survival in patients with NPC. It may improve the predictive value of the TNM classification and help to identify patients likely to benefit from more aggressive therapeutic regimens.

## Background

Nasopharyngeal carcinoma (NPC) is a head and neck cancer that shows a distinct endemic distribution, with a high prevalence in southern China and surrounding areas [1]. Radiotherapy is the mainstay treatment modality for NPC. With the advent of intensity-modulated radiation therapy (IMRT), local control for NPC has improved significantly, and distant metastasis is the main cause of treatment failure at present [2].

Currently, the prognosis of patients with NPC is evaluated primarily with the TNM staging system, which is the most important prognostic factor. However, a discrepancy sometimes exists between actual treatment outcomes and TNM classifications. This happens because metastasis is predisposed to occur in some patients with early-stage cancers, whereas a relatively long-term survival may be observed in certain patients with locoregionally advanced diseases [3,4]. For the former group of patients, radiotherapy alone may be insufficient and is likely to end with failure; for the latter group of patients, aggressive treatment such as chemoradiotherapy may result in overtreatment, which causes toxicity, decreases quality of life, and even increases the risk of non-cancer death [5]. This phenomenon highlights limitations of the TNM staging system, which does not take functional factors into consideration. Hence, to improve the potential for individualized treatment, it is reasonable to consider other factors in addition to the TNM staging system for more accurate and practical prognostication for NPC [6].

Platelets play important roles in physiological and pathological pathways and have been linked to tumor metastasis and other oncological processes. Tumor-related thrombocytosis is a common phenomenon in patients with solid cancers, and it has been identified as an unfavorable prognostic factor in lung cancer, breast cancer, and many other malignancies [7]. To our knowledge, however, few studies have investigated the characteristics and outcomes of patients with NPC and thrombocytosis. Previously, we analyzed 1,582 patients with NPC and determined that a platelet count greater than 300 × 10^9^/L before radiotherapy was an unfavorable prognostic factor for survival and distant metastasis [8]. However, the outdated 2002 Union for International Cancer Control/American Joint Committee on Cancer (UICC/AJCC) staging system was used in that study, and the association between platelet count and the TNM classification was not fully investigated, providing no evidence of whether platelet count could be used together with the TNM staging system to improve prognostic prediction. Also, as we know, patients with advanced-stage NPC often require chemoradiotherapy, and chemotherapy may inhibit marrow function and affect platelet count before radiotherapy. Thus, in this study, we enlarged the sample size, restaged all patients according to the newest 7th edition of the UICC/AJCC staging system, and obtained platelet count data before treatment (chemoradiotherapy or radiotherapy) to accurately investigate the significance and prognostic value of pretreatment platelet count for treatment outcomes compared with established prognostication systems. Moreover, adding chemotherapy to radiotherapy helps to reduce the risk of metastasis in patients with NPC. Thus, we performed matched-pair analysis between patients who received chemoradiotherapy and those who received radiotherapy alone to assess the real effect of these treatments and to aid in planning individualized treatment.

## Patients and Methods

### Patient selection and clinical staging

We conducted a retrospective study of 2,669 patients who were newly diagnosed with NPC without distant metastasis between January 2001 and December 2004 at the Sun Yat-sen University Cancer Center (SYSUCC). Inclusion criteria were as follows: (1) newly diagnosed, histologically proven NPC; (2) no distant metastasis; and (3) receiving radical radiotherapy. Exclusion criteria were as follows: (1) lack of pretreatment platelet count records (n = 12) and (2) concomitant diseases that may affect platelet count, such as autoimmune disease, history of blood transfusion, liver cirrhosis, and severe hypertension (n = 31). Thus, a total of 2,626 patients were included and their data were analyzed in this study. Computed tomography and/or magnetic resonance imaging was essential for disease staging before treatment, and all cases were restaged according to the 7th edition of the UICC/AJCC staging system [9]. This study was approved by the Hospital Ethics Committee of SYSUCC. Written consent was waived as it was a retrospective study, and verbal consent was obtained via telephone.

### Platelet count measurement

Pretreatment platelet count was measured at baseline within 7 days before treatment (chemoradiotherapy or radiotherapy) for all patients. A platelet count greater than 300 × 10^9^/L was defined as thrombocytosis, according to the normal range of platelet counts in our institution (100 × 10^9^/L to 300 × 10^9^/L).

### Grouping and matching

**Figure 1:**
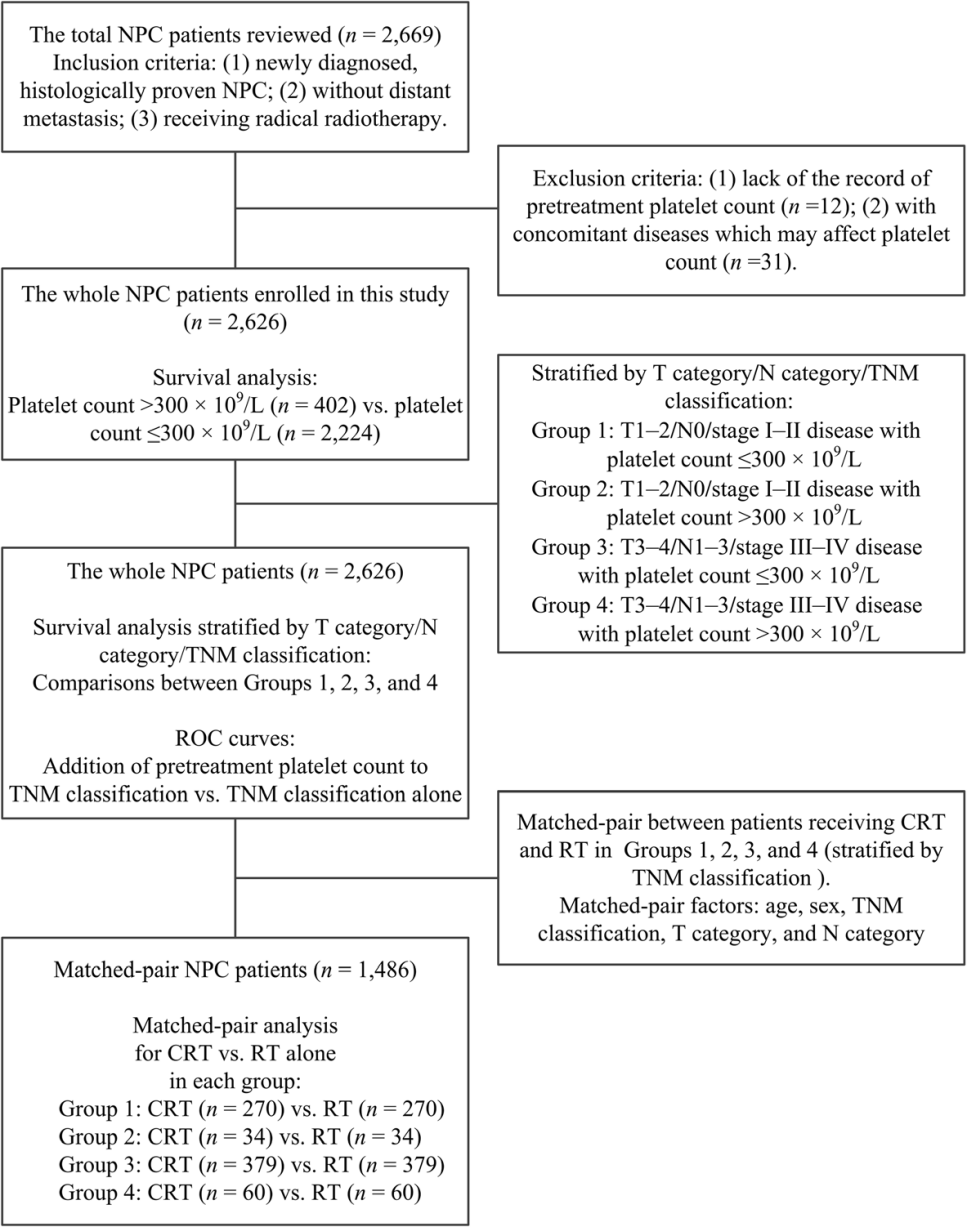
**Flowchart of study design.** NPC, nasopharyngeal carcinoma; ROC, receiver operating characteristic; CRT, chemoradiotherapy; RT, radiotherapy.

The flowchart of our study design is shown in Figure 1. We first performed survival analysis based on pretreatment platelet count in all 2,626 patients with NPC. Then, patients were categorized into 4 groups to analyze the prognostic value of pretreatment platelet count when stratified by T category, N category, and TNM classification as follows: Group 1, T1–2/N0/stages I–II disease with platelet count ≤300 × 10^9^/L; Group 2, T1–2/N0/stages I–II disease with platelet count >300 × 10^9^/L; Group 3, T3–T4/N1–3/stages III–IV disease with platelet count ≤300 × 10^9^/L; and Group 4, T3–T4/N1–3/stages III–IV disease with platelet count >300 × 10^9^/L.

To balance other prognostic factors and validate the real association between therapeutic regimens and treatment outcomes in patients in the 4 groups, we performed a one-to-one match between patients receiving chemoradiotherapy and radiotherapy based on the randomization pairing principle for matched-pair analysis. Matching was performed according to age, sex, TNM classification, T category, and N category. We generated 743 matched pairs of patients, with 270 pairs in Group 1, 34 pairs in Group 2, 379 pairs in Group 3, and 60 pairs in Group 4.

### Radiotherapy

All patients were treated with definitive-intent radiotherapy with high-energy, 6 MV to 8 MV X-ray by linear accelerator, including 2,400 patients (91.4%) treated with two-dimensional conformal radiotherapy (2D-CRT), 61 (2.3%) treated with three-dimensional conformal radiotherapy (3D-CRT), and 165 (6.3%) treated with IMRT.

Opposing lateral facial-cervical fields were used in the 2D-CRT to cover the nasopharynx and upper cervical lymphatic drainage region, with one lower anterior cervical field to cover the lower cervical region. After 36 to 40 Gy of radiation, opposing lateral preauricular fields were used for the primary region, and anterior split neck fields were used for the cervical region. The primary tumor was irradiated to a dose of 60 to 78 Gy. The dose of prophylactic irradiation for patients treated with 2D-CRT was 50 to 54 Gy to the prophylactic areas. For 3D-CRT, the total prescribed dose was 66 to 72 Gy to the gross tumor volume of the nasopharynx (GTVnx), 60 to 70 Gy to the gross tumor volume of the involved metastatic lymph nodes (GTVnd), 60 Gy to clinical target volumes-1 (CTV-1, the GTVnx and an additional 5- to 10-mm margin), and 50 to 54 Gy to the prophylactic irradiating region (CTV-2). For IMRT, the target definition and delineation were the same as described above for 3D-CRT. The prescription dose was 68 Gy to the GTVnx, 60 to 64 Gy to the GTVnd of the neck, 60 Gy to the CTV-1, and 54 Gy to the CTV-2.

### Chemotherapy

Of 2,626 patients, 1,216 (46.3%) received neoadjuvant, concurrent, or adjuvant chemotherapy. The majority of the patients (774 of 1,100; 70.3%) with stage III or IV NPC (classified as T3–4 and/or N2–3 disease) received chemotherapy. Neoadjuvant or adjuvant chemotherapy consisted of cisplatin and 5-fluorouracil or taxanes every 3 weeks for 2 or 3 cycles. Concurrent chemotherapy consisted of either cisplatin plus 5-fluorouracil or cisplatin alone given weekly or on weeks 1, 4, and 7 of radiotherapy.

### Follow-up

After completing treatment, patients were followed up every 3 months for the first 3 years and the intervals gradually increased to 6 to 12 months after 3 years. February 1st, 2011 was the date of last follow-up.

The primary endpoint was overall survival (OS), and the secondary endpoints were locoregional recurrence–free survival (LRFS) and distant metastasis–free survival (DMFS). OS was calculated as the period from the start of treatment to death from any cause. LRFS and DMFS were calculated as the period from the start of treatment to the first occurrence of locoregional and distant failure, respectively.

### Statistical analysis

All analyses were performed using SPSS software, version 19.0. The Chi-square test was used to compare categorical variables. The rates of OS, LRFS, and DMFS were estimated by means of the Kaplan-Meier method and differences were compared by using the log-rank test. Multivariate analysis using a Cox proportional hazards model was used to test the independent significance of different variables by backward elimination. The covariates entering into the multivariable analysis included host factors (age and sex), tumor factors (T and N categories), and treatment factor (chemoradiotherapy or radiotherapy) for the entire patient cohort and the 4 patient groups, and host factors and tumor factors for matched pairs of patients.

Receiver operating characteristic (ROC) curve analysis was used to evaluate the predicted validity of pretreatment platelet count, based on the method of Hanley and McNeil [10,11]. The comparison of areas under the curve (AUCs) was made by *Z* test, and a two-sided *P* value of < 0.05 was considered statistically significant.

## Results

### Patient characteristics

The baseline characteristics of the 2,626 patients analyzed in this study are shown in Table 1. The median age was 46 years (range, 11–78 years). Among the 2,626 patients, 2,387 (90.9%) had undifferentiated non-keratinizing carcinoma, 222 (8.4%) had differentiated non-keratinizing carcinoma, and 17 (0.6%) had other types of cancer.

**Table 1:** **Baseline characteristics in the entire cohort of 2,626 patients with nasopharyngeal carcinoma (NPC)**

**Patient characteristic**	**No. of patients [** ***n*** **(%)]**	**PLT count [** ***n*** **(%)]**		
		**≤300 × 10** ^**9**^ **/L**	**>300 × 10** ^**9**^ **/L**	***P*** **value**
Age (years)				0.202
≤45	1,308 (49.8)	1,096 (49.3)	212 (52.7)	
>45	1,318 (50.2)	1,128 (50.7)	190 (47.3)	
Sex				<0.001
Male	2,015 (76.7)	1,740 (78.2)	275 (68.4)	
Female	611 (23.3)	484 (21.8)	127 (31.6)	
TNM classification				0.021
I	157 ( 6.0)	134 ( 6.0)	23 ( 5.7)	
II	899 (34.2)	778 (35.0)	121 (30.1)	
III	1,036 (39.5)	882 (39.7)	154 (38.3)	
IV	534 (20.3)	430 (19.3)	104 (25.9)	
T category				<0.001
T1	516 (19.6)	449 (20.2)	67 (16.7)	
T2	1,010 (38.5)	870 (39.1)	140 (34.8)	
T3	655 (24.9)	557 (25.0)	98 (24.4)	
T4	445 (16.9)	348 (15.6)	97 (24.1)	
N category				0.616
N0	669 (25.5)	566 (25.4)	103 (25.6)	
N1	1,081 (41.2)	920 (41.4)	161 (40.0)	
N2	773 (29.4)	647 (29.1)	126 (31.3)	
N3	103 (3.9)	91 ( 4.1)	12 (3.0)	
Treatment				0.652
CRT	1,216 (46.3)	1,034 (46.5)	182 (45.3)	
RT	1,410 (53.7)	1,190 (53.5)	220 (4.7)	
Total	2,626	2,223	402	

PLT, platelet; CRT, chemoradiotherapy; RT, radiotherapy.

The median duration of follow-up was 81.6 months (range, 1.4–124.7 months). Of all 2,626 patients, 493 (18.8%) developed locoregional failure, 368 (14.0%) developed distant metastasis, and 774 (29.5%) died. For the entire cohort, the 5-year OS, LRFS, and DMFS rates were 76.7%, 84.4%, and 86.7%, respectively.

### Characteristics and survival analysis in the entire patient cohort

Pretreatment platelet count ranged from 58 × 10^9^/L to 572 × 10^9^/L with a median value of 224 × 10^9^/L. Among all 2,626 patients, 402 (15.3%) had a platelet count greater than 300 × 10^9^/L. Thrombocytosis occurred more frequently in female patients than in male patients (20.8% vs. 13.6%, *P* < 0.001), in patients with advanced T-category (T3–4) lesions than in patients with early T-category (T1–2) lesions (17.7% vs. 13.6%, *P* < 0.001), and in patients with locoregionally advanced NPC (stages III–IV) than in patients with early-stage NPC (stages I–II) (16.4% vs. 13.6%, *P* = 0.021) (Table 1). No significant differences in age, N category, or treatment were found.

Univariate analysis indicated that platelet count was a significant prognostic factor for OS (*P* < 0.001) and DMFS (*P* < 0.001), but not for LRFS (*P* = 0.264). In multivariate analysis, platelet count was an independent unfavorable prognostic factor for OS [hazard ratio (HR) = 1.810, 95% confidence interval (CI) = 1.531–2.140, *P* < 0.001] and DMFS (HR = 1.873, 95% CI = 1.475–2.379, *P* < 0.001).

### Subgroup analysis in patients stratified by T category/N category/TNM classification

**Figure 2:**
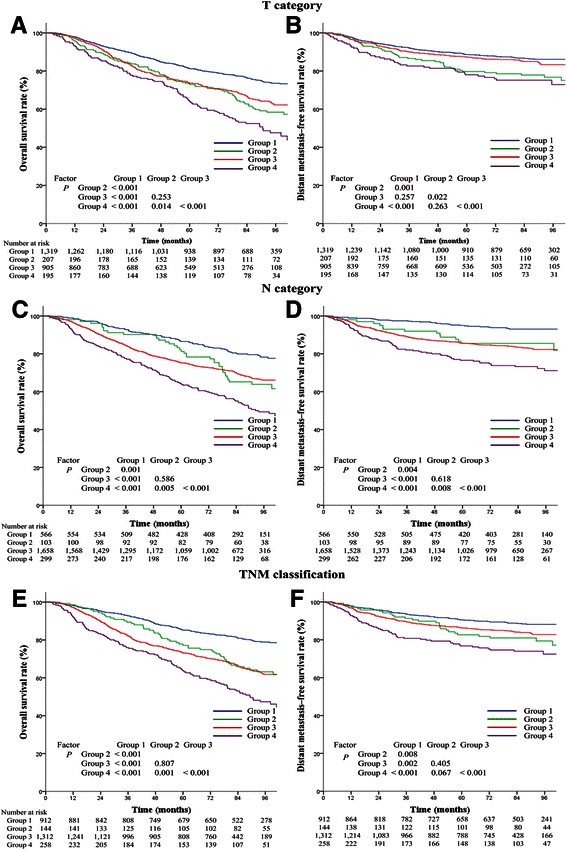
**Survival analysis based on pretreatment platelet count (≤300 × 10** ^**9**^ **/L and >300 × 10** ^**9**^ **/L) in all 2,626 patients with nasopharyngeal carcinoma stratified by T category/N category/TNM classification. A**, overall survival curves for the 4 groups stratified by T category. **B**, distant metastasis–free survival curves for the 4 groups stratified by T category. **C**, overall survival curves for the 4 groups stratified by N category. **D**, distant metastasis–free survival curves for the 4 groups stratified by N category. **E**, overall survival curves for the 4 groups stratified by TNM classification. **F**, distant metastasis–free survival curves for the 4 groups stratified by TNM classification. Group 1: T1–2/N0/stages I–II disease with platelet count ≤300 × 10^9^/L; Group 2: T1–2/N0/stages I–II disease with platelet count >300 × 10^9^/L; Group 3: T3–4**/**N1–3**/**stages III–IV disease with platelet count ≤300 × 10^9^/L; Group 4: T3–4/N1–3/stages III–IV disease with platelet count >300 × 10^9^/L.

Stratified by T category, patients in Group 1 and Group 2 had 5-year OS rates of 81.3% and 72.9% (*P* < 0.001), respectively, and 5-year DMFS rates of 88.6% and 79.8% (*P* = 0.001), respectively; in patients in Group 3 and Group 4, the 5-year OS rates were 73.7% and 64.3% (*P* < 0.001), respectively (Figures 2A), and the 5-year DMFS rates were 87.3% and 78.0% (*P* < 0.001), respectively (Figures 2B). Stratified by N category, patients in Group 1 and Group 2 had 5-year OS rates of 86.5% and 81.3% (*P* = 0.001), respectively, and a 5-year DMFS rates of 95.0% and 85.5% (*P* = 0.004), respectively; in patients in Group 3 and Group 4, the 5-year OS rates were 75.3% and 64.2% (*P* < 0.001), respectively (Figures 2C), and the 5-year DMFS rates were 85.6% and 76.6% (*P* < 0.001), respectively (Figures 2D). For LRFS, no significant difference was found between patients with different platelet counts after T**/**N category stratification. Multivariate analysis showed that a high platelet count was an independent unfavorable prognostic factor for OS and DMFS not only in patients with advanced T/N categories (Group 3 and Group 4) but also in patients with early T**/**N categories (Group 1 and Group 2) (all *P* ≤ 0.001).

Stratified by TNM classification, patients in Group 1 and Group 2 had 5-year OS rates of 85.2% and 77.1% (*P* < 0.001), respectively, and 5-year DMFS rates of 90.5% and 82.6% (*P* = 0.008), respectively; in patients in Group 3 and Group 4, the 5-year OS rates were 73.3% and 63.9% (*P* < 0.001), respectively (Figure 2E), and the 5-year DMFS rates were 86.3% and 76.8% (*P* < 0.001), respectively (Figure 2F). Overall, the 5-year OS and DMFS rates of patients with different platelet counts were significantly different when stratified by TNM classification (Group 1 vs. Group 2; Group 3 vs. Group 4). However, no significant difference was found between Group 2 and Group 3. For LRFS, no significant difference was observed between patients with different platelet counts when stratified by TNM classification. In multivariate analysis, a high platelet count was an independent unfavorable prognostic factor for OS and DMFS not only in patients with locoregionally advanced NPC (Group 3 and Group 4) but also in patients with early-stage NPC (Group 1 and Group 2) (all *P* ≤ 0.01; Table 2).

**Table 2:** **Multivariate analyses of prognostic factors for patients with early-stage and locoregionally advanced NPC**

**Endpoint**	**Variable** ^**a**^	**HR**	**95% CI**	***P*** **value**
**Early-stage NPC (** ***n*** **= 1,057)**				
Overall survival	Age (>45 vs. ≤45 years)	1.586	1.213–2.075	0.001
	Sex (female vs. male)	1.409	1.019–1.949	0.038
	T category (T2 vs. T1)	1.352	1.014–1.803	0.040
	N category (N1 vs. N0)	2.326	1.732–3.124	<0.001
	PLT count (>300 × 10^9^/L vs. ≤300 × 10^9^/L)	2.057	1.510–2.804	<0.001
Locoregional recurrence–free survival	N category (N1 vs. N0)	1.566	1.053–2.328	0.027
Distant metastasis–free survival	Age (>45 vs. ≤45 years)	1.387	0.970–1.984	0.073
	T category (T2 vs. T1)	1.899	1.249–2.888	0.003
	N category (N1–3 vs. N0)	2.532	1.677–3.824	<0.001
	PLT count (>300 × 10^9^/L vs. ≤300 × 10^9^/L)	1.788	1.167–2.739	0.008
**Locoregionally advanced NPC (** ***n*** **= 1,569)**				
Overall survival	Age (>45 vs. ≤45 years)	1.657	1.397–1.966	<0.001
	Gender (female vs. male)	1.452	1.163–1.813	0.001
	PLT count (>300 × 10^9^/L vs. ≤300 × 10^9^/L)	1.716	1.407–2.093	<0.001
Locoregional recurrence–free survival	Age (>45 vs. ≤45 years)	1.541	1.238–2.037	<0.001
	Gender (female vs. male)	1.561	1.259–1.888	0.001
Distant metastasis–free survival	N category (N1–3 vs. N0)	2.014	1.331–3.047	0.001
	PLT count (>300 × 10^9^/L vs. ≤300 × 10^9^/L)	1.859	1.393–2.481	<0.001

^**a**^The parameters listed in this column were included in the multivariate analysis using Cox proportional hazards model by backward elimination. PLT, platelet; CRT, chemoradiotherapy; RT, radiotherapy; HR, hazard ratio; CI, confidence interval.

### The validity of adding pretreatment platelet count to TNM classification for predicting survival in the entire patient cohort

**Figure 3:**
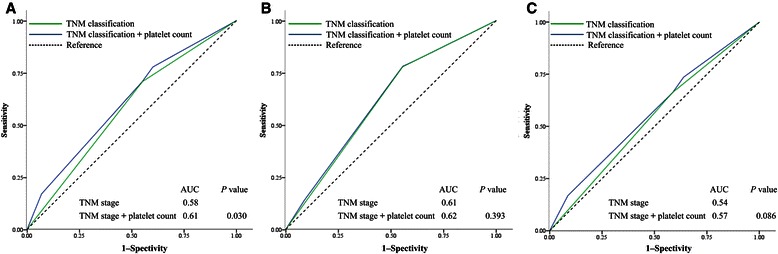
**Receiver operating characteristic (ROC) curves for survival prediction with TNM classification alone (stages I–II and III–IV) or with the addition of pretreatment platelet count ( ≤300 × 10** ^**9**^ **/L and >300 × 10** ^**9**^ **/L) to TNM classification in the 2,626 patients with nasopharyngeal carcinoma. A**, overall survival. **B**, locoregional recurrence–free survival. **C**, distant metastasis–free survival. The *P* value was the comparison of area under the curves (AUCs) between the addition of platelet count to TNM classification and TNM classification alone.

ROC curves were used to compare the validity of adding pretreatment platelet count (≤300 × 10^9^/L and >300 × 10^9^/L) to TNM classification (stages I–II and III–IV) for prognostic prediction. For OS, the AUC was 0.58 for TNM classification alone and 0.61when platelet count was added to TNM classification (*P* = 0.030) (Figure 3A). The results revealed that the addition of platelet count to TNM classification was superior to TNM classification alone in predicting survival for patients with NPC. No significant improvement in predicting locoregional recurrence was observed with the addition of platelet count to TNM classification (*P* = 0.393) (Figure 3B). For DMFS, adding platelet count to TNM classification had a tendency to improve prediction of distant metastasis (*P* = 0.086) (Figure 3C).

### Prognostic value of chemoradiotherapy in matched pairs of patients in the 4 groups stratified by TNM classification

**Figure 4:**
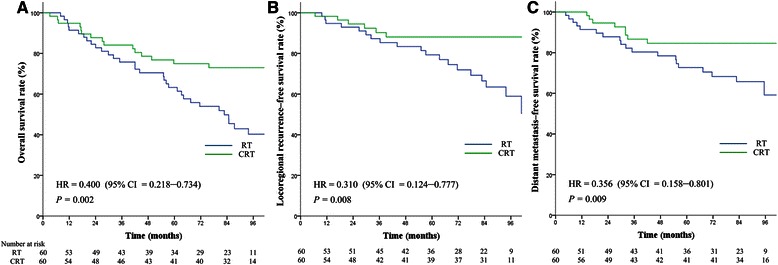
**Survival analysis based on treatment [chemoradiotherapy (CRT) and radiotherapy (RT)] in matched pairs of patients with stages III–IV nasopharyngeal carcinoma and platelet count >300 × 10** ^**9**^ **/L. A**, overall survival. **B**, locoregional recurrence–free survival. **C**, distant metastasis–free survival. Hazards ratios (HRs) were calculated using the unadjusted Cox proportional hazards model. *P* values were calculated using the unadjusted log-rank test.

The baseline characteristics of the 743 matched pairs of patients are shown in Table 3. In Group 1, the 5-year OS, LRFS, and DMFS rates for patients receiving radiotherapy and chemoradiotherapy were 80.1% vs. 80.8% (*P* = 0.623), 91.6% vs. 90.2% (*P* = 0.284), and 87.6% vs. 85.0% (*P* = 0.198). In Group 2, the 5-year OS, LRFS, and DMFS rates for patients receiving radiotherapy and chemoradiotherapy were 59.8% vs. 73.5% (*P* = 0.337), 90.0% vs. 96.0% (*P* = 0.378), and 65.1% vs. 87.2% (*P* = 0.068). In Group 3, the 5-year OS, LRFS, and DMFS rates for patients receiving radiotherapy and chemoradiotherapy were 70.7% vs. 78.3% (*P* = 0.085), 79.4% vs. 84.7% (*P* = 0.175), and 88.2% vs. 88.9% (*P* = 0.772). There was no significant difference among the treatment outcomes for the aforementioned 3 groups. In Group 4, the 5-year OS, LRFS, and DMFS rates for patients receiving radiotherapy and chemoradiotherapy were 63.3% vs. 75.0% (*P* = 0.002), 79.3% vs. 88.1% (*P* = 0.008), and 72.7% vs. 84.6% (*P* = 0.009), indicating a significant difference for all treatment outcomes (Figure 4). Cox regression also showed that chemoradiotherapy significantly improved OS (HR = 0.416, 95% CI = 0.226–0.765, *P* = 0.005), LRFS (HR = 0.327, 95% CI = 0.130–0.820, *P* = 0.017), and DMFS (HR = 0.366, 95% CI = 0.162–0.825, *P* = 0.015) in Group 4.

**Table 3:** **Baseline characteristics in matched pairs of patients with NPC**

**Patient characteristic**	**Group 1 (** ***N*** **= 270 pairs)**		**Group 2 (** ***N*** **= 34 pairs)**		**Group 3 (** ***N*** **= 379 pairs)**		**Group 4 (** ***N*** **= 60 pairs)**	
	**RT [** ***n*** **(%)]**	**CRT [** ***n*** **(%)]**	**RT [** ***n*** **(%)]**	**CRT [** ***n*** **(%)]**	**RT [** ***n*** **(%)]**	**CRT [** ***n*** **(%)]**	**RT [** ***n*** **(%)]**	**CRT [** ***n*** **(%)]**
Age (years)								
≤45	138 (51.1)	138 (51.1)	18 (52.9)	18 (52.9)	171 (45.1)	171 (45.1)	31 (51.7)	31 (51.7)
>45	132 (48.9)	132 (48.9)	16 (47.1)	16 (47.1)	208 (54.9)	208 (54.9)	29 (48.3)	29 (48.3)
Sex								
Male	71 (26.3)	71 (26.3)	8 (23.5)	8 (23.5)	79 (20.8)	79 (20.8)	15 (25.0)	15 (25.0)
Female	199 (73.7)	199 (73.7)	26 (76.5)	26 (76.5)	300 (79.2)	300 (79.2)	45 (75.0)	45 (75.0)
TNM classification								
I	4 (1.5)	4 (1.5)	0 (0)	0 (0)	0 (0)	0 (0)	0 (0)	0 (0)
II	266 (98.5)	266 (98.5)	34(100.0)	34 (100.0)	0 (0)	0 (0)	0 (0)	0 (0)
III	0 (0)	0 (0)	0 (0)	0 (0)	293 (77.3)	293 (77.3)	39 (65.0)	39 (65.0)
IV	0 (0)	0 (0)	0 (0)	0 (0)	86 (22.7)	86 (22.7)	21 (35.0)	21 (35.0)
T category								
T1	72 (26.7)	72 (26.7)	9 (26.5)	9 (26.5)	38 (10.0)	38 (10.0)	3 (5.0)	3 (5.0)
T2	198 (73.3)	198 (73.3)	25 (73.5)	25 (73.5)	92 (24.3)	92 (24.3)	12 (20.0)	12 (20.0)
T3	0 (0)	0 (0)	0 (0)	0 (0)	167 (44.1)	167 (44.1)	24 (40.0)	24 (40.0)
T4	0 (0)	0 (0)	0 (0)	0 (0)	82 (21.6)	82 (21.6)	21 (35.0)	21 (35.0)
N category								
N0	28 (10.4)	28 (10.4)	3 (8.8)	3 (8.8)	87 (23.0)	87 (23.0)	10 (16.7)	10 (16.7)
N1	242 (89.6)	242 (89.6)	31 (91.2)	31 (91.2)	103 (27.2)	103 (27.2)	20 (33.3)	20 (33.3)
N2	0 (0)	0 (0)	0 (0)	0 (0)	185 (48.8)	185 (48.8)	30 (50.0)	30 (50.0)
N3	0 (0)	0 (0)	0 (0)	0 (0)	4 (1.1)	4 (1.1)	0 (0)	0 (0)

CRT, chemoradiotherapy; RT, radiotherapy. Group 1: early-stage disease with platelet count ≤300 × 10^9^/L; Group 2: early-stage disease with platelet count >300 × 10^9^/L; Group 3: locoregionally advanced disease with platelet count ≤300 × 10^9^/L; Group 4: locoregionally advanced disease with platelet count >300 × 10^9^/L.

## Discussion

Pretreatment platelet count is a common and practical laboratory examination. This study demonstrates that thrombocytosis was associated with poor OS (HR = 1.810, 95% CI = 1.531–2.140, *P* < 0.001) and increased the incidence of distant metastasis (HR = 1.873, 95% CI = 1.475–2.379, *P* < 0.001) in patients with NPC. Thrombocytosis remained an independent unfavorable prognostic factor in subgroup analysis stratified by T category/N category/TNM classification. Thrombocytosis was more frequent in patients with advanced-stage disease, occurring in 13.6% (144 of 1,056) of patients with early-stage NPC (stages I–II) and in 16.4% (257 of 1,570) of patients with locoregionally advanced NPC (stages III–IV) (*P* = 0.021).

Increased platelet count can be commonly observed in malignancies (10% to 57%) [12]. The cause of tumor-related thrombocytosis still remains unclear but interleukin-6 (IL-6) is thought to be responsible, as it mediates tumor-related production of thrombopoietin, a granulocyte-macrophage colony-stimulating factor [13]. Accumulating evidence shows that increased platelet count enhances the metastatic potential of tumor cells by facilitating immune evasion, promoting extravasation, and impeding natural killer cells [4]. The association between thrombocytosis and TNM classification indicates an interaction between platelets and tumor cells in tumor progression. Platelets can secrete a number of proangiogenic cytokines such as vascular endothelial growth factor [14] and thymidine phosphorylase [15]. These platelet-derived factors regulate angiogenesis and cell proliferation, which are associated with extensive tumor invasion and poor response to chemoradiotherapy [16,17]. Advanced cancer can further induces the production and activation of platelets through tumor-associated tissue factors such as IL-6, thrombin, and ADP, thus creating a vicious circle [18]. Similar association between thrombocytosis and TNM classification was also found in other solid cancers [19-21]. Along with proangiogenic cytokines, platelets also promote angiogenesis directly via integrins by mediating cell-to-cell adhesion [22] and increasing the risk of thrombosis through the hypercoagulable state developed in cancer patients [23] and therefore result in poor prognosis.

Another major objective of our study was to verify the prognostic value of both pretreatment platelet count and disease stage. Although advanced-stage disease is usually associated with poor local control and survival, patients with different platelet counts and tumors in the same TNM classification may have different prognoses. In the stratified analysis, patients with tumors in the same TNM classification (early-stage or advanced) and different platelet counts had significantly different OS and DMFS. Notably, there was no significant difference in OS and DMFS between patients with early-stage NPC with thrombocytosis (Group 2) and those with locoregionally advanced NPC without thrombocytosis (Group 3). This reiterates the limitations of the current TNM staging system, which is based on anatomic involvement and does not accurately reflect the level of malignancy. Increased pretreatment platelet count may reflect increased tumor burden and result in poor prognosis beyond the TNM staging system. The ROC curve results suggest that the prediction of survival can be significantly improved by adding pretreatment platelet count to the current TNM staging system (*P* = 0.030). Also, adding platelet count to TNM classification had a strong tendency to improve the prediction of distant metastasis (*P* = 0.086). Such improvements to the prognostic system would facilitate better stratification such that patients with predicted poorer prognosis could receive more aggressive treatment protocols.

From the results of matched-pair analysis between patients receiving different therapeutic regimens, we can see that chemoradiotherapy did not improve any treatment outcome in patients with early-stage NPC with or without thrombocytosis (Group 1 and Group 2) although there was a strong tendency to reduce the risk of distant metastasis in Group 2 (*P* = 0.078). For patients with locoregionally advanced NPC without thrombocytosis (Group 3), chemoradiotherapy had a strong tendency to improve survival (*P* = 0.093), and for patients with locoregionally advanced NPC with thrombocytosis (Group 4), chemoradiotherapy improved all treatment outcomes. One major goal of adding chemotherapy to radiotherapy in patients with NPC is to reduce micrometastases. The results above indicate that pretreatment platelet count can serve as a useful tool to identify patients at high risk of metastasis and poor survival, who could benefit from more aggressive treatment such as chemoradiotherapy. Patients with early-stage NPC without thrombocytosis may gain less or even no benefit from aggressive therapeutic strategies, which means chemoradiotherapy probably results in overtreatment and the adverse effects outweigh the advantages. In contrast, patients with early-stage NPC with thrombocytosis may benefit from chemoradiotherapy, as it has a tendency to reduce the risk of metastasis. The insignificant improvement in survival may result from the relatively early-stage and small matched-pair samples. As patients with advanced-stage NPC with thrombocytosis may gain the most benefit from chemoradiotherapy, it is possible that these patients may require a more intensive systemic treatment. However, for patients with advanced-stage NPC without thrombocytosis, clinicians using current chemoradiotherapy strategies should monitor for overtreatment and modulate the treatment plan as necessary, as these patients stand to gain less benefit. Moreover, with respect to thrombocytosis, anticoagulants may be combined with radiotherapy or chemoradiotherapy to improve treatment outcomes. A meta-analysis of 11 studies demonstrated that anticoagulants could significantly improve OS in cancer patients [24].

This study has several limitations. This is a retrospective study, in which the results might be affected by some confounding factors and the bias could not be neglected. Also, the absolute difference between AUCs was relatively small, indicating that platelet count alone was insufficient for establishing an excellent predictive model. Additional predictive factors are needed to improve the model’s performance. Moreover, although previous meta-analyses indicated that both neoadjuvant chemotherapy and concurrent chemotherapy with or without adjuvant chemotherapy could improve OS in patients with locoregionally advanced NPC [25,26], the demonstrated superiority of chemoradiotherapy to radiotherapy alone could be a mixed effect of the combined modalities, which is hard to distinguish. In addition, only a small proportion of patients (6.3%) received IMRT. As IMRT can improve local control significantly, we presume that the predictive ability of pretreatment platelet count for metastasis and survival would be more apparent in patients receiving IMRT, and these patients may gain more benefit from the addition of chemotherapy if thrombocytosis is present. Despite these limitations, this is a single-institution study with a large sample size that specifically reports the prognostic value of pretreatment platelet count in association with the TNM classification \and therapeutic regimens in patients with NPC. The results of this study could aid further refinement of the current TNM staging system. Furthermore, pretreatment platelet count, which can be measured easily and at low cost, may aid in developing individualized treatment strategies to improve the prognosis of patients with NPC and therefore represents an important therapeutic prospect.

## Conclusions

Pretreatment platelet count is a useful indicator for metastasis and survival in NPC patients. It helps to improve the prediction accuracy of the TNM staging system. Also, it is a useful tool to identify patients who may benefit from more aggressive therapeutic regimens.

## References

[1] Jemal A, Bray F, Center MM, Ferlay J, Ward E, Forman D (2011). Global cancer statistics. CA Cancer J Clin.

[2] Lai SZ, Li WF, Chen L, Luo W, Chen YY, Liu LZ (2011). How does intensity-modulated radiotherapy versus conventional two-dimensional radiotherapy influence the treatment results in nasopharyngeal carcinoma patients?. Int J Radiat Oncol Biol Phys.

[3] Wang HY, Sun BY, Zhu ZH, Chang ET, To KF, Hwang JS (2011). Eight-signature classifier for prediction of nasopharyngeal [corrected] carcinoma survival. J Clin Oncol.

[4] Li G, Gao J, Tao YL, Xu BQ, Tu ZW, Liu ZG (2012). Increased pretreatment levels of serum LDH and ALP as poor prognostic factors for nasopharyngeal carcinoma. Chin J Cancer.

[5] Talmi YP, Horowitz Z, Bedrin L, Wolf M, Chaushu G, Kronenberg J (2002). Quality of life of nasopharyngeal carcinoma patients. Cancer.

[6] Lee AW, Ng WT, Chan LK, Chan OS, Hung WM, Chan CC (2012). The strength/weakness of the AJCC/UICC staging system (7th edition) for nasopharyngeal cancer and suggestions for future improvement. Oral Oncol.

[7] Buergy D, Wenz F, Groden C, Brockmann MA (2012). Tumor-platelet interaction in solid tumors. Int J Cancer.

[8] Gao J, Zhang HY, Xia YF (2013). Increased platelet count is an indicator of metastasis in patients with nasopharyngeal carcinoma. Tumor Biol.

[9] Pan J, Xu Y, Qiu S, Zong J, Guo Q, Zhang Y, et al. A comparison between the Chinese 2008 and the 7th edition AJCC staging systems for nasopharyngeal carcinoma. Am J Clin Oncol. 2013. [Epub ahead of print] 10.1097/COC.0b013e31828f5c96 23608833

[10] Zweig MH, Campbell G (1993). Receiver-operating characteristic (ROC) plots: a fundamental evaluation tool in clinical medicine. Clin Chem.

[11] Hanley JA, McNeil BJ (1982). The meaning and use of the area under a receiver operating characteristic (ROC) curve. Radiology.

[12] Sierko E, Wojtukiewicz MZ (2004). Platelets and angiogenesis in malignancy. Semin Thromb Hemost.

[13] Kaser A, Steurer W, Offner FA, Theurl I, Molnar C, Atkins MB (2001). Interleukin-6 stimulates thrombopoiesis through thrombopoietin: role in inflammatory thrombocytosis. Blood.

[14] Möhle R, Green D, Moore MA, Nachman RL, Rafii S (1997). Constitutive production and thrombin-induced release of vascular endothelial growth factor by human megakaryocytes and platelets. Proc Natl Acad Sci U S A.

[15] Okamoto E, Kase S, Kaibara N (2001). Thymidine phosphorylase expression causes both the increase of intratumoral microvessels and decrease of apoptosis in human esophageal carcinomas. Pathol Int.

[16] Shimada H, Hoshino T, Okazumi S, Matsubara H, Funami Y, Nabeya Y (2002). Expression of angiogenic factors predicts response to chemoradiotherapy and prognosis of oesophageal squamous cell carcinoma. Br J Cancer.

[17] Shimada H, Takeda A, Shiratori T, Nabeya Y, Okazumi S, Matsubara H (2002). Prognostic significance of serum thymidine phosphorylase concentration in esophageal squamous cell carcinoma. Cancer.

[18] Nash GF, Turner LF, Scully MF, Kakkar AK (2002). Platelets and cancer. Lancet Oncol.

[19] Chen MH, Chang PM, Chen PM, Tzeng CH, Chu PY, Chang SY (2009). Prognostic significance of a pretreatment hematologic profile in patients with head and neck cancer. J Cancer Res Clin Oncol.

[20] Erdemir F, Kilciler M, Bedir S, Ozgok Y, Coban H, Erten K (2007). Clinical significance of platelet count in patients with renal cell carcinoma. Urol Int.

[21] Lu CC, Chang KW, Chou FC, Cheng CY, Liu CJ (2007). Association of pretreatment thrombocytosis with disease progression and survival in oral squamous cell carcinoma. Oral Oncol.

[22] Pipili-Synetos E, Maragoudakis ME (1998). Evidence that platelets promote tube formation by endothelial cells on matrigel. Brit J Pharmacol.

[23] van Doormaal FF, Raskob GE, Davidson BL, Decousus H, Gallus A, Lensing AWA (2009). Treatment of venous thromboembolism in patients with cancer: subgroup analysis of the matisse clinical trials. Thromb Haemostasis.

[24] Kuderer NM, Lyman GH (2007). A meta-analysis and systematic review of the efficacy and safety of anticoagulants as cancer treatment—impact on survival and bleeding complications. Cancer.

[25] Langendijk JA, Leemans CR, Buter J, Berkhof J, Slotman BJ (2004). The additional value of chemotherapy to radiotherapy in locally advanced nasopharyngeal carcinoma: a meta-analysis of the published literature. J Clin Oncol.

[26] OuYang PY, Xie C, Mao YP, Zhang Y, Liang XX, Su Z (2013). Significant efficacies of neoadjuvant and adjuvant chemotherapy for nasopharyngeal carcinoma by meta-analysis of published literature-based randomized, controlled trials. Ann Oncol.

